# CBP and p300 Histone Acetyltransferases Contribute to Homologous Recombination by Transcriptionally Activating the *BRCA1* and *RAD51* Genes

**DOI:** 10.1371/journal.pone.0052810

**Published:** 2012-12-20

**Authors:** Hideaki Ogiwara, Takashi Kohno

**Affiliations:** Division of Genome Biology, National Cancer Center Research Institute, Chuo-ku, Tokyo, Japan; St. Georges University of London, United Kingdom

## Abstract

Histone acetylation at DNA double-strand break (DSB) sites by CBP and p300 histone acetyltransferases (HATs) is critical for the recruitment of DSB repair proteins to chromatin. Here, we show that CBP and p300 HATs also function in DSB repair by transcriptionally activating the *BRCA1* and *RAD51* genes, which are involved in homologous recombination (HR), a major DSB repair system. siRNA-mediated depletion of CBP and p300 impaired HR activity and downregulated *BRCA1* and *RAD51* at the protein and mRNA levels. Chromatin immunoprecipitation assays showed that CBP and p300 bind to the promoter regions of the *BRCA1* and *RAD51* genes, and that depletion of CBP and/or p300 reduces H3 and H4 acetylation and inhibits binding of the transcription factor E2F1 to these promoters. Depletion of CBP and p300 impaired DNA damage-induced phosphorylation and chromatin binding of the single-strand DNA-binding protein RPA following BRCA1-mediated DNA end resection. Consistent with this, subsequent phosphorylation of CHK1 and activation of the G2/M damage checkpoint were also impaired. These results indicate that the HATs CBP and p300 play multiple roles in the activation of the cellular response to DSBs.

## Introduction

Chromosomal DNA and histones form a highly condensed structure known as chromatin. Access to chromosomal DNA by transcription factors and DNA repair proteins is regulated by chromatin remodeling, including histone modification and nucleosomal alteration [1]. Acetylation of histones by histone acetyltransferases (HATs) is a critical chromatin modification required for transcription and double-strand break (DSB) repair. We recently reported that the homologous HATs CBP and p300 facilitate DSB repair by acetylating histones H3 and H4 at DSB sites [2], [3], [4], supporting previous findings that modifications of histones H3 and H4 are critical for DSB repair [2], [5]. Indeed, CBP and p300 are required for the recruitment to DSB sites of KU70/80, which are key proteins involved in non-homologous end joining (NHEJ), a major DSB repair system [6]. Together, these studies suggest that histone acetylation by the CBP and p300 HATs facilitates chromatin remodeling at DSB sites, and that the resulting “relaxed” chromatin permits the recruitment and accumulation of DNA repair proteins at these sites.

CBP and p300 are well known to function as transcriptional co-activators by producing “relaxed” chromatin accessible to transcription factors through the acetylation of histones H3 and H4 at gene promoter regions (Karamouzis et al., 2007); however, no previous reports have addressed the contribution of CBP/p300 to the transcriptional activation of genes involved in DSB repair. In fact, we previously showed that the expression levels of genes involved in NHEJ are not significantly affected by CBP or p300 [2], [3], [4]. Here, we demonstrate that CBP and p300 activate the transcription of the *BRCA1* and *RAD51* genes, which are key regulators of homologous recombination (HR), the other major DSB repair system. In DSB-induced HR, which occurs after DSB recognition by the MRE11-RAD50-NBS1 (MRN) complex, BRCA1 contributes to DNA end resection at DSBs as a member of the CtIP-MRN-BRCA1 complex, allowing the phosphorylation of RPA (RPA70/RPA32/RPA16) and binding of phosphorylated RPA to single-stranded DNA (ssDNA) [7], [8], [9], [10]. RAD51 plays a major role in the pairing of homologous DNAs and strand transfer in HR [11]. RAD51 is recruited to DSB sites via phosphorylation by CHK1, which is itself phosphorylated by ATR upon activation by RPA-bound ssDNA [12]. CHK1 phosphorylation also activates the DNA damage checkpoint, arresting the cell cycle to allow sufficient time for DNA repair to take place [13], [14]. Expression of the *RAD51* and *BRCA1* genes is strictly regulated by E2F transcriptional factors in a cell cycle-dependent manner [15], [16], [17]; however, the involvement of HATs in the transcriptional activation of these two genes has not been reported to date. In this study, we show that CBP and p300 contribute to the transcriptional activation of the *BRCA1* and *RAD51* genes, resulting in the promotion of DSB-induced HR and activation of the DNA damage checkpoint.

## Materials and Methods

### Cell Lines

H1299 and A549 human lung cancer cells were cultured in RPMI 1640 supplemented with 10% fetal bovine serum (FBS) at 37°C with 5% CO2. HeLa and HeLa DR-GFP (kindly gifted from Dr. Komatsu) [18] human cervical carcinoma cells were cultured in DMEM supplemented with 10% FBS. H1299, A549, and HeLa cells were obtained from the American Type Culture Collection (ATCC).

### Chemicals and siRNA

Camptothecin (CPT) and neocarzinostatin (NCS) were purchased from Sigma. ON-TARGETplus smart pool siRNA duplexes were obtained from Dharmacon. The specific siRNAs used were siNT (non-targeting) (L-001810-10), siCBP (L-003477-00), sip300 (L-003486-00), siBRCA1 (L-003461-00), and siBRCA2 (L-003462-00).

### Antibodies

Antibodies against the following proteins were used: RAD51 (Abcam, ab213), 53BP1 (Abcam, ab36823), RPA32 (Calbiochem; NA18), phospho-RPA32-pS4/S8 (Bethyl A300-245A-1), RPA70 (Calbiochem, NA13), Mre11 (Novus, NB100-142), RAD50 (Genetex, GTX70228), NBS1 (BD, 611870), H2B (Millipore, 07-371), γH2AX (Millipore, 05-636 and Cell Signaling Technologies, #9718), BRCA1 (Santa Cruz Biotechnology, sc-6954), BRCA2 (Calbiochem, OP95), ATM (Epitomics, 1549-1), phospho-ATM-S1981 (Epitomics, 2152-1), CHK2 (Epitomics, 3428-1), phospho-CHK2-T68 (Cell Signaling Technologies, #2661), CHK1 (Sigma, C9358), phospho-Chk1-S345 (Cell Signaling Technologies, 2348), β actin (Sigma, A5441), XRCC4 (Abcam, ab12069), DNA-PKcs, (Calbiochem, NA57), CBP (Santa Cruz Biotechnology, sc-369 and sc-7300), p300 (Santa Cruz Biotechnology, sc-585 and sc-48343), E2F1 (Santa Cruz Biotechnology, sc-251), E2F4 (Santa Cruz Biotechnology, sc-866), KU70 (Santa Cruz Biotechnology, sc-9033), KU80 (Santa Cruz Biotechnology, sc-9034), ORC2 (Santa Cruz Biotechnology, sc-28742), Histone H3 (Active Motif, 39164), acetylated H4 (K5/8/12/16) (Millipore, 06-866), acetylated H3 (K18) (Millipore, 07–354), and acetylated H2B (K5/12/15/20) (Millipore, 07–373).

### HR assay

HeLa cells (1×10^5^ per well) carrying the DR-GFP reporter were grown in 12-well plates and either transfected with the I-*Sce*I expression plasmid pCBASce (0.5 μg) or mock-transfected. Transfections were performed using Lipofectamine LTX reagent (Invitrogen). Cells were then grown for 48 hr prior to analysis. Cells were harvested by trypsinization, washed with PBS, resuspended in 200 μl PBS, passed through a 40 μm cell strainer, and analyzed using a Guava flow cytometer (Millipore). Flow cytometry profiles were generated using the GuavaSoft 2.2 software (Becton Dickinson). For quantitative PCR analysis, genomic DNA was purified from cells using the DNeasy Blood & Tissue Kit (Qiagen), and 20 ng of DNA was subjected to quantitative PCR to quantify the uncut and repaired DNAs using an ABI 7900HT Fast Real-Time PCR System analyzer (Applied Biosystems). PCR was performed for 40 cycles consisting of denaturation at 95°C for 15 sec, and annealing and extension at 60°C for 60 sec using a Power SYBR Green PCR Master Mix (Applied Biosystems). Primer sets used for quantification of the uncut, repaired, and *GAPDH* gene body regions are as follows: uncut region (DRGFP-Uncut-FW1: 5′-TGCTGTCTCATCATTTTGGCA-3′, DRGFP-Uncut-RV1: 5′-CCCTGTTATCCCTAGCCGGA-3′), repaired region (DRGFP-HRR-FW1: 5′-CGTGTCCGGCGAGGG-3′, DRGFP-HRR-RV1: 5′-ATGTTGTGGCGGATCTTGAAG-3′) and the *GAPDH* gene body region (GAPDH-RT-FW2: 5′-ATGCTGAGTGTACAAGCGTTTTCT-3′, GAPDH-RT-RV2: 5′-CACTATGCCACCCCAGGAAT-3′). For each sample, the proportions of uncut and repaired products were calculated as ratios to the amount of *GAPDH* gene body region (internal control). Uncut product ratios were shown as ratios of the pCBASce plasmid untransfected sample. Repaired product ratios were shown as ratios to the siNT (control siRNA)-treated sample.

### Immunofluorescence analysis

Cells were grown on glass coverslips, fixed with 2% (w/v) paraformaldehyde in PBS for 15 min at room temperature, and then permeabilized with 0.3% (v/v) Triton X-100 for 15 min at room temperature. After fixation, cells were washed three times with PBS and blocked with blocking buffer (BB: 1% bovine serum albumin (BSA), 0.1% Triton X-100 in PBS) for 10 min. Cells were incubated with primary antibody (diluted in BB) for 1 hr at room temperature, washed with PBS, and counter-stained for 1 hr at room temperature with Alexa Fluor 488 goat anti-mouse, Alexa Fluor 488 goat anti-rabbit, Alexa Fluor 568 goat anti-mouse, or Alexa Fluor 568 goat anti-rabbit secondary antibodies (Molecular Probes) diluted in BB. Cells were then washed three times with PBS and stained with DAPI (0.4 μg/ml in PBS) to visualize DNA. Coverslips were mounted onto glass slides using Prolong Gold mounting agent (Invitrogen). Confocal images were acquired using an inverted microscope (Carl Zeiss) equipped with a 100× oil lens and the ZEN 2008 software (Carl Zeiss).

### Immunoblot analysis

Cells were harvested, washed with PBS, and lysed in 100 μl NETN300 buffer (20 mM Tris-HCl, pH 7.5, 300 mM NaCl, 0.5% NP-40, 1 mM EDTA) containing 1 mM PMSF, a proteinase inhibitor cocktail, and a phosphatase inhibitor cocktail (Roche Diagnostics). Whole cell lysates were mixed with SDS sample buffer. To isolate insoluble chromatin, cells were lysed in NETN300 buffer containing complete protease inhibitor cocktail (Roche), incubated for 30 min on ice, and centrifuged at 4°C for 10 min at 14,000 rpm. The resulting insoluble chromatin was sheared twice by sonication for 15 sec. Lysates were separated by SDS-PAGE, transferred to PVDF membranes, and immunoblotted with antibodies as indicated. Membranes were blocked overnight with TBS containing 0.1% Tween 20 and 0.5% BSA and probed with primary antibodies. After washing with TBS containing 0.1% Tween 20, the membranes were incubated with horseradish peroxidase-conjugated anti-mouse or anti-rabbit secondary antibodies and visualized using an enhanced chemiluminescence reagent (GE Healthcare).

### Cell cycle analysis

Cells were treated with the indicated agents and collected by trypsinization 1 hr later. Cells were washed in PBS and then fixed in ice-cold 70% ethanol with gentle vortexing. Fixed cells were re-centrifuged and passed through a 40 μm cell strainer, incubated with PBS containing 200 μg/ml RNase A and 5 μg/ml propidium iodide (PI; Sigma-Aldrich), and analyzed using a Guava flow cytometer.

### Quantification of mRNA

Cells were plated and transfected with siRNA using Lipofectamine RNAMAX (Invitrogen). At 48 hr after transfection, mRNAs and cDNAs were extracted using the TaqMan Gene Expression Cells-to-CT Kit (Applied Biosystems). Aliquots were subjected to quantitative PCR using gene-specific primer/probe sets (RAD51: Hs00153418_m1; BRCA1: Hs01556193_m1; GAPDH: 4326317E) (Applied Biosystems) and an ABI 7900HT Fast Real-Time PCR System (Applied Biosystems). PCR was performed for 45 cycles (denaturation at 95°C for 15 sec, annealing and extension at 60°C for 60 sec) using TaqMan Gene Expression Master Mix (Applied Biosystems). For each sample, BRCA1 and RAD51 mRNA levels were normalized against the level of GAPDH mRNA; BRCA1/GAPDH and RAD51/GAPDH ratios were further normalized against the corresponding ratio from the siNT sample.

### Chromatin immunoprecipitation (ChIP) assay

Cells (2×10^6^) were plated in 100 mm dishes and transfected with siRNA using Lipofectamine RNAMAX. At 48 hr after transfection, cells were treated with 1% formaldehyde for 10 min at room temperature to crosslink proteins to DNA, followed by the addition of glycine (0.125 M) to stop the crosslinking. Cells were collected with a cell scraper and washed in ice-cold PBS. The ChIP assay was performed using a ChIP-IT Express Enzymatic kit (Active Motif). Briefly, resuspended cells were subjected to DNA shearing for 10 min at 37°C, followed by the addition of EDTA to stop the reaction. An aliquot of each sample was set aside as the input control, while the remaining portion was subjected to immunoprecipitation at 4°C overnight. The cross-links were then reversed in reverse cross-linking buffer for 15 min at 95°C, and the samples were de-proteinized for 1 hr at 37°C using Proteinase K. Aliquots were subjected to quantitative PCR using gene-specific primer sets (RAD51 promoter: 5′-CCCCCGGCATAAAGTTTGA-3′ and 5′-GCTTTCAGAATTCCCGCCA-3′; BRCA1 promoter: 5′-TTTCGTATTCTGAGAGGCTGCTG-3′ and 5′-ATTTATCTGTAATTCCCGCGCTT-3′; *GAPDH* gene body: 5′-ATGCTGAGTGTACAAGCGTTTTCT-3′, and 5′-CACTATGCCACCCCAGGAAT-3′) and an ABI 7900HT Fast Real-Time PCR System. PCR was performed for 45 cycles (denaturation at 95°C for 15 sec, annealing and extension at 60°C for 60 sec) using Power SYBR Green PCR Master Mix (Applied Biosystems). The enrichment of proteins was measured by two values, normalized and relative ChIP enrichment. The former is used to show the degree of enrichment following a specific pulldown with anti-p300 and anti-CBP antibodies. The latter is used to show the decrease/increase in enrichment following gene knockdown at test and control regions. To calculate normalized ChIP enrichment, first ChIP enrichment at the test sites (the *BRCA1* or *RAD51* promoters and the *GAPDH* gene body) was calculated as the fraction of the total input DNA that was pulled down by the specific antibody or the non-specific control IgG. Then, normalized ChIP enrichment at the test sites was calculated by dividing the ChIP enrichment of the specific antibody by that of the non-specific control IgG. The relative ChIP enrichment following siRNA treatment was calculated by dividing the ChIP enrichment at the *BRCA1* or *RAD51* promoter and the *GAPDH* gene body in the siRNA-treated sample by the ChIP enrichment at the *BRCA1* or *RAD51* promoter in the sample treated with non-targeting siRNA (siNT). The antibodies used in the ChIP assay were as follows: CBP (sc-369), p300 (sc-585), E2F1 (sc-251), E2F4 (sc-866), acetylated H4 (K5/8/12/16) (06–866), and acetylated H3 (K18) (07–354).

### DNA damage checkpoint analysis

Cells were exposed to 3 Gy ionizing radiation (IR) (^60^Co source) after exposure to the indicated agents for 1 hr. Cells were harvested 1 hr after IR exposure, washed twice with ice-cold PBS, fixed, permeabilized with 70% ethanol, and stored at −20°C for at least 2 hr. Fixed cells were blocked with blocking buffer (0.5% BSA in PBS) for 10 min at room temperature. Cells were then stained with an anti-phospho-Histone H3 (Ser10) antibody at 1∶25 dilution in blocking buffer for 1 hr at room temperature. Washes were carried out three times in PBS. For secondary staining, cells were incubated for 1 hr at room temperature with FITC-conjugated donkey anti-mouse IgG antibody (Jackson ImmunoResearch) diluted 1∶100. After secondary antibody staining, cells were washed three times, incubated with 5 μg/mL PI and 200 μg/mL RNase I in PBS, and analyzed using a Guava flow cytometer. The resulting data were analyzed using the GuavaSoft 2.2 software (Becton Dickinson).

### Clonogenic assay

H1299, HeLa, and A549 cells were transfected with siRNA, incubated for 48 hr, and then trypsinized, counted, and plated in 6-well dishes (500–1000 cells/dish). Cells were grown for 5 hr before treatment with CPT. Following a 14 day recovery period, cells were fixed in 50% (v/v) methanol/0.01% (w/v) crystal violet solution for 5 min. Survival points were assayed in triplicate. The number of colonies derived from treated cells was normalized against the number of colonies derived from untreated cells.

## Results

### Depletion of CBP and p300 suppresses the activation of homologous recombination

**Figure 1:**
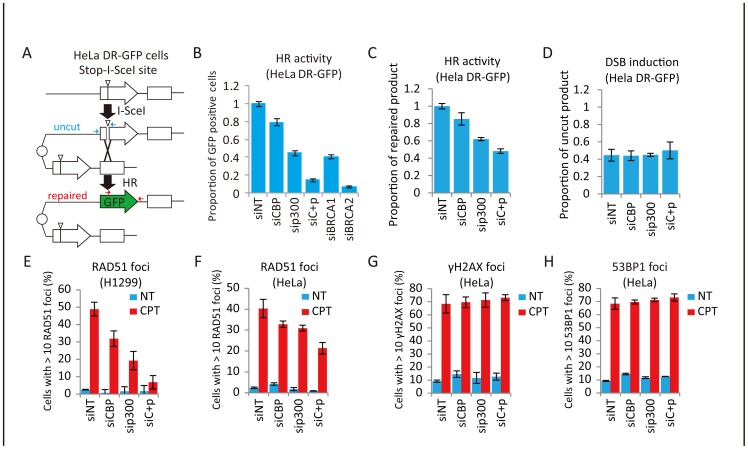
Involvement of CBP and p300 in HR. (**A**) HR Assay design. I-*Sce*I sites are indicated by white triangle heads. The locations of the PCR primers used for quantitative PCR to monitor DSB introduction by I-*Sce*I (uncut DNA) and the subsequent repair (repaired DNA) are indicated by blue and red arrows, respectively. (B) Suppression of *I-Sce*I-induced HR upon CBP and p300 depletion. HeLa DR-GFP cells, after transfection for 48 hr with non-targeting (siNT), CBP (siCBP), p300 (sip300), CBP+p300 (siC+p), BRCA1 (siBRCA1), or BRCA2 (siBRCA2) siRNAs, were transfected with an *I-Sce*I expression plasmid or mock-transfected. Forty-eight hours after transfection, cells were harvested and assayed for GFP expression by flow cytometry. Data represent the mean ± SD. (**C, D**) Assessment of DSB generation and DNA repair. (C) Proportion of repaired product 48 hr after transfection of the I-*Sce*I expression plasmid. The proportion of repaired product detected after targeting siRNA treatment is expressed as a ratio to that detected after non-targeting siRNA (siNT) treatment. (D) Proportion of uncut product at the I-*Sce*I site 24 hr after transfection of the I-*Sce*I expression plasmid. The proportion of uncut product is expressed as a ratio to the amount of uncut product present before I-*Sce*I expression plasmid transfection. Data represent the mean ± SD. (**E, F, G, H**) Suppression of CPT-induced foci formation upon depletion of CBP and/or p300. HeLa or H1299 cells were transfected with non-targeting (siNT), CBP (siCBP), p300 (sip300), or CBP+p300 (siC+p) siRNAs for 48 hr. Cells were then treated with DMSO (NT) or 1 μM camptothecin (CPT), fixed and processed for immunofluorescence detection of RAD51 (E, H1299; F, HeLa), γH2AX (G, HeLa), or 53BP1 (H, HeLa). Data represent the mean ± SD.

The possible involvement of CBP and p300 in DSB-induced HR was investigated *in vivo* by using a GFP-based chromosomal assay in which a DSB is generated by expression of the *I-Sce*I endonuclease, whose recognition site is integrated in the GFP gene such that digestion disrupts the gene (**Figure** **1A**) [19]. Single knockdown of either CBP or p300 by siRNA treatment reduced the proportion of GFP-positive cells, and double knockdown of CBP and p300 caused a greater reduction similar to that caused by BRCA1 and BRCA2 ablation (**Figure** **1B** **and Figure** **S1A**). The proportion of repaired products, but not that of the cut products, was reduced after ablation of CBP and/or p300 (**Figure** **1C–D**), indicating that down-regulation of these HATs inhibits the repair activity but not DSB generation. To examine the molecular processes underlying the HR deficiency in these cells, the formation of nuclear foci associated with the DSB response and DNA repair was investigated. Single- and double-knockdown cells showed a significant reduction in the CPT- or IR-induced formation of RAD51 foci (**Figure** **1E–F** **and Figure** **S1C-E**), but not of γH2AX and 53BP1 foci (**Figure** **1G–H**), which are markers for DSB formation. These results indicated that CBP and p300 contribute to DSB-induced HR.

### Down-regulation of *BRCA1* and *RAD51* expression by depletion of CBP and p300

**Figure 2:**
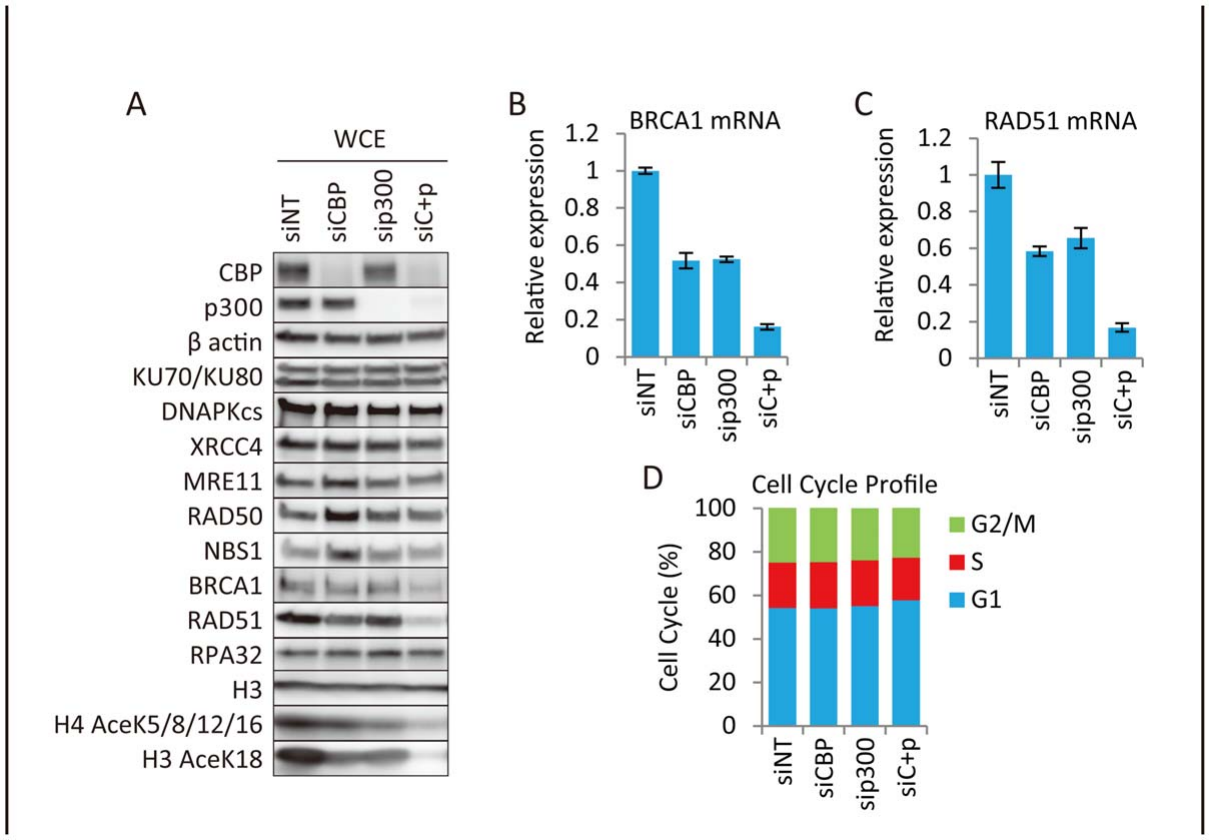
Involvement of CBP and p300 in the transcription of the *BRCA1* and *RAD51* genes. (**A**) Down-regulation of BRCA1 and RAD51 proteins upon depletion of CBP and/or p300. H1299 cells were transfected for 48 hr with non-targeting (siNT), CBP (siCBP), p300 (sip300), or CBP+p300 (siC+p) siRNAs. The cells were harvested and whole cell extracts were subjected to immunoblotting. (**B, C**) Reduction of *BRCA1* and *RAD51* transcripts in CBP- and p300-depleted cells. H1299 cells were transfected for 48 hr with non-targeting (siNT), CBP (siCBP), p300 (sip300), or CBP+p300 (siC+p) siRNAs. Cells were harvested and subjected to quantitative real-time PCR for the detection of *BRCA1* (B) and *RAD51* (C) mRNAs. Expression levels were normalized against the levels of *GAPDH* mRNA. Data represent the mean ± SD. (**D**) H1299 cells were transfected for 48 hr with non-targeting (siNT), CBP (siCBP), p300 (sip300), or CBP+p300 (siC+p) siRNAs, and stained with propidium iodide (PI). The percentage of cells in each cell cycle phase was determined by FACS. Percentages of cells in G1, S, and G2/M are shown.

CBP and p300 function as transcriptional activators by acetylating histones at gene promoter regions [20]. Therefore, we examined the expression of representative genes encoding proteins involved in HR and that of other proteins involved in the recognition/repair of DSB by siRNA-mediated depletion of CBP and p300 in H1299 cells. Protein levels of BRCA1 and RAD51, but not those of RPA32, significantly decreased upon double knockdown of CBP and p300, whereas the levels of proteins involved in DSB recognition (MRE11, RAD50, and NBS1) and NHEJ (KU70, KU80, DNA-PKcs, and XRCC4) were not affected (**Figure** **2A**). Depletion of CBP and p300 in these experiments was verified by monitoring changes in their protein levels as well as the acetylation status of histones (**Figure** **2A**). The down-regulation of BRCA1 and RAD51 upon depletion of CBP and p300 was confirmed in other cell lines (HeLa and A549) (**Figure** **S2A–B**).

Quantitative RT-PCR showed that *BRCA1* and *RAD51* mRNA levels decreased upon CBP/p300 knockdown, suggesting that CBP and p300 act as transcriptional co-activators for these two genes (H1299 cells in **Figure** **2B–C** **and** HeLa and A549 cells in **S2C–F**). *RAD51* and *BRCA1* transcription levels are lowest during G0/G1 and highest in the S and G2 phases of the cell cycle [15], [16]. Therefore, to exclude the possibility that the observed decreases in *RAD51* and *BRCA1* transcripts were due to changes in the cell cycle profile, cell cycle progression was examined after depletion of CBP and p300. Knockdown of either of these genes did not affect cell cycle profiles (**Figure** **2D**), suggesting that the decrease in the levels of *BRCA1* and *RAD51* mRNA was caused by transcriptional suppression induced by CBP and p300 depletion. Double knockdown of CBP and p300 caused a significantly higher reduction in BRCA1 and RAD51 protein expression and HR activity than the depletion of either gene alone (**Figure** **1** **and** **2**). These results suggested that CBP and p300 redundantly contribute to transcriptional activation of the *BRCA1* and *RAD51* genes.

### Depletion of CBP and p300 impairs histone acetylation and E2F1 binding at the *BRCA1* and *RAD51* promoters

**Figure 3:**
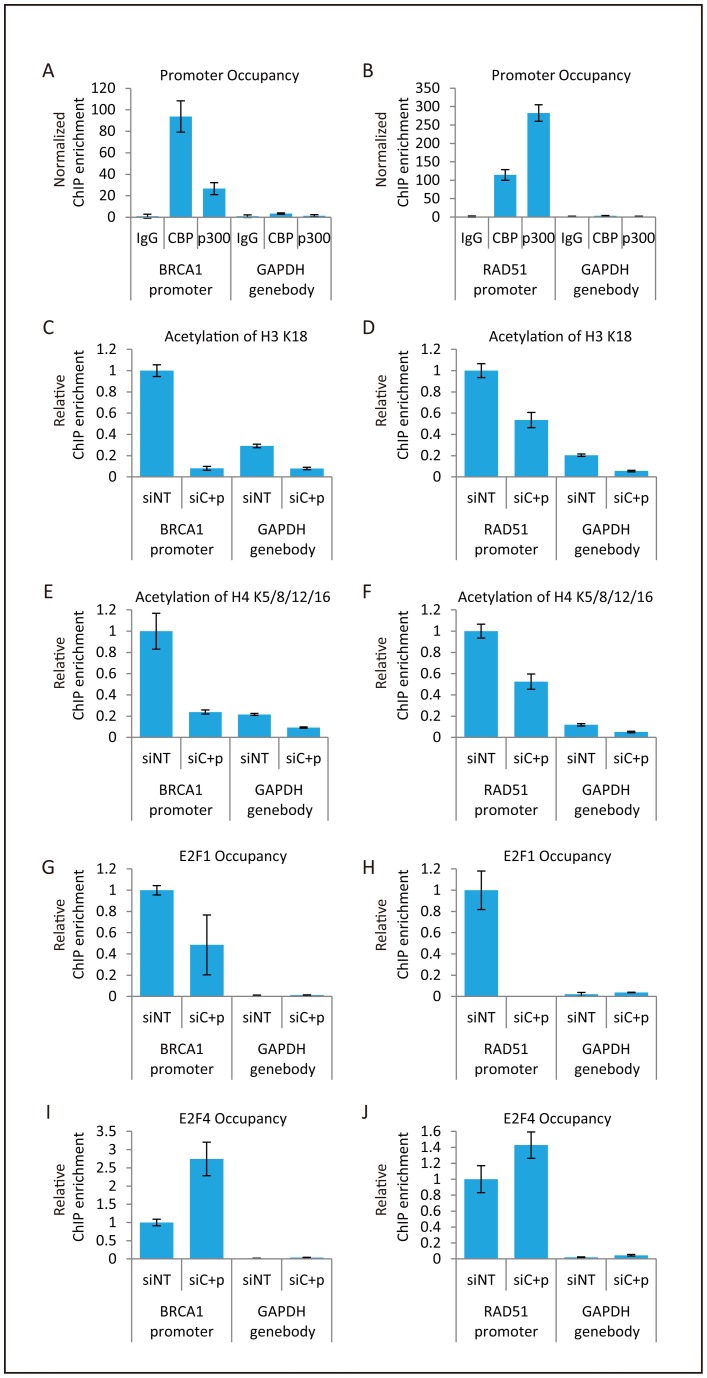
Histone acetylation and E2F1 binding at the *BRCA1* and *RAD51* promoter regions. (**A, B**) CBP and p300 recruitment to the *BRCA1* and *RAD51* promoter regions. H1299 cells were subjected to ChIP using either control IgG or antibodies against CBP and p300. Immunoprecipitated DNAs were subjected to quantitative PCR for the detection of the promoter region of *BRCA1* (A) or *RAD51* (B) and for the detection of the non-promoter region of *GAPDH*. Normalized ChIP enrichment was calculated to show the degree of enrichment following pulldown with anti-p300 and anti-CBP antibodies. First, ChIP enrichment at the test sites (*BRCA1* or *RAD51* promoter and the *GAPDH* gene body) was calculated as the fraction of total input DNA that was pulled down by the specific antibody or the non-specific control IgG. Then, normalized ChIP enrichment at the test sites was calculated by dividing the ChIP enrichment of the specific antibody by that of the non-specific control IgG. Data represent mean values ± SD. (**C–J**) Impaired histone acetylation and changes in E2F1 and E2F4 binding at *BRCA1* and *RAD51* promoter regions caused by knockdown of CBP and p300. H1299 cells pre-transfected with non-targeting (siNT) or CBP and p300 (siC+p) siRNAs were subjected to ChIP assays. DNA immunoprecipitated with anti-acetylated H3 K18 (H3 AceK18) (C, D), anti-acetylated H4 (H4 AceK5/8/12/16) (E, F), anti-E2F1 (G, H), or anti-E2F4 (I, J) antibodies was subjected to quantitative PCR to detect the promoter regions of *BRCA1* (C, E, G, I) and *RAD51* (D, F, H, J), and the gene body region of *GAPDH*. Relative ChIP enrichment was calculated to show the decrease/increase in enrichment at these sites following gene knockdown. The relative ChIP enrichment was calculated by dividing the ChIP enrichment at the *BRCA1* or *RAD51* promoter and the *GAPDH* gene body region in the siRNA-treated sample by the ChIP enrichment at the *BRCA1* or *RAD51* promoter in the siNT-treated sample. Data represent the mean ± SD.

Acetylation of lysines in the N-terminal tails of histones at gene promoter regions favors active transcription [21], [22], and CBP and p300 contribute to such lysine acetylation [20]. Therefore, we next performed ChIP analysis to investigate CBP and p300 binding to the promoter regions of the *BRCA1* and *RAD51* genes. Quantitative PCR analysis showed that enrichment of CBP and p300 at the *BRCA1* and *RAD51* promoter regions, which contain E2F transcription factor binding sites [17], was higher than at the *GAPDH* gene body region, i.e., a unrelated gene region (**Figure** **3A–B**). These results indicated that CBP and p300 directly bind to the *BRCA1* and *RAD51* promoter regions.

p300 acetylates lysines 14 and 18 of histone H3, and lysines 5 and 8 of histone H4 *in vitro* [23], [24], [25]. In addition, lysine 18 of histone H3 is acetylated by CBP and p300 *in vivo* [26]. Furthermore, in this study, we confirmed that the depletion of CBP and p300 reduces the acetylation levels of histone H3 and H4 (**Figure** **2A**). Taken together, these findings strongly suggest that CBP and p300 function as HATs for histones H3 and H4. Therefore, we next examined the involvement of CBP and p300 in the acetylation of histones H3 and H4 at the *BRCA1* and *RAD51* promoter regions by ChIP analysis using anti-acetylated histone antibodies. The acetylation levels of histone H3 K18 and histone H4 K5/K8/K12/K16 were higher at the *BRCA1* and *RAD51* promoter regions than at the *GAPDH* gene body region, and were decreased upon depletion of CBP and p300 (**Figure** **3C–F**).

We next examined whether knockdown of CBP and p300 alters the binding of E2F family proteins to the *BRCA1* and *RAD51* promoter regions. In particular, we examined the activating transcription factor E2F1 and the repressive factor E2F4 [27]. Similar to CBP and p300, E2F1 and E2F4 were enriched at the *BRCA1* and *RAD51* promoter regions compared to the *GAPDH* gene body region (**Figure** **3G–J**). Knockdown of CBP and p300 decreased E2F1 binding and increased E2F4 binding. Our results showing the localization of CBP and p300, together with the changes in histone H3/H4 acetylation and E2F1/E2F4 binding upon depletion of CBP and p300, strongly suggest that CBP and p300 function as HATs to activate *BRCA1* and *RAD51* transcription.

### Depletion of CBP and p300 impairs the DSB-induced RPA-CHK1 pathway

**Figure 4:**
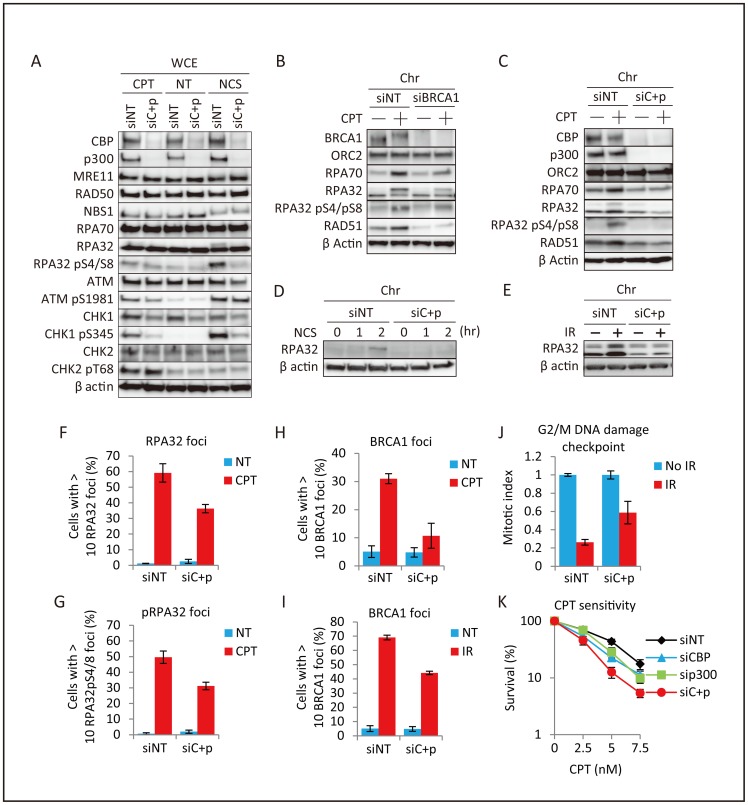
Impairment of RPA foci formation and the DNA damage checkpoint in CBP and p300 depleted cells. (**A**) Effects of CBP and p300 depletion on DSB-induced RPA and CHK1 activation. H1299 cells were pre-transfected with non-targeting (siNT) or CBP and p300 (siC+p) siRNA for 48 hr, and treated with DMSO (NT), 1 μM camptothecin (CPT), or 500 ng/ml neocarzinostatin (NCS) for 2 hr. Whole cell extracts were subjected to immunoblot analysis. (**B, C**) Decreased DSB-induced chromatin binding of RPA proteins. Chromatin enriched fractions from H1299 cells pre-transfected for 48 hr with non-targeting (siNT), BRCA1 (B), or CBP and p300 (siC+p) (C) siRNAs and treated with DMSO (NT) or 1 μM camptothecin (CPT) for 2 hr were subjected to immunoblot analysis. (**D, E**) Decreased DNA damage-induced chromatin binding of RPA proteins in H1299 cells. Chromatin enriched fractions from cells pre-transfected for 48 hr with non-targeting (siNT) or CBP+p300 (siC+p) siRNA were treated or not treated (NT) with (D) 500 ng/ml neocarzinostatin (NCS) for 1 or 2 hr, or (E) incubated for 3 hr after 50 Gy ionizing irradiation (IR) and subjected to immunoblot analysis. (**F, G**) Decreased DSB-induced RPA32 foci formation. H1299 cells were transfected with non-targeting (siNT) or CBP and p300 (siC+p) siRNA for 48 hr, treated with DMSO (NT) or 1 μM camptothecin (CPT) for 3 hr, and subjected to immunofluorescence analysis. The percentages of cells with RPA32 foci (F) or phosphorylated RPA32 (pS4/S8) foci (G) are shown. Data represent the mean ± SD. (**H, I**) CPT- and IR-induced formation of BRCA1 foci. H1299 cells were transfected with non-targeting (siNT) or CBP and p300 (siC+p) siRNA for 48 hr, treated with 1 μM camptothecin (CPT) for 3 hr (H) or 10 Gy ionizing irradiation (IR) for 4 hr (I), and subjected to immunofluorescence analysis. The percentages of cells showing >10 BRCA1 foci are shown. Data represent the mean ± SD. (**J**) Suppression of the G2/M DNA damage checkpoint. H1299 cells were pre-transfected for with non-targeting (siNT) or CBP and p300 (siC+p) siRNA for 48 hr. The cells were then irradiated (IR, 3 Gy) for 1 hr or not irradiated (No IR), and stained with anti-phospho-histone H3 antibody and propidium iodide (PI). The percentage of mitotic cells (mitotic index) was determined by flow cytometry. Data represent the mean ± SD. (**K**) Colony formation of H1299 cells treated with CPT. H1299 cells were pre-transfected for with non-targeting (siNT) or CBP and p300 (siC+p) siRNA for 48 hr. siRNA-treated cells were replated and incubated for 10 days. Survival is expressed as a percentage representing the number of colonies in treated samples relative to the number in DMSO-treated samples.

BRCA1 forms a complex with CtIP and MRE11/RAD50/NBS1, which promotes DSB end resection to generate RPA-coated ssDNA [28], [29]. Depletion of BRCA1 reduces the formation of ssDNA, which is necessary for the recruitment of both RPA and RAD51; thus, BRCA1 functions upstream of RAD51 in DSB repair [28], [30]. Consistent with these facts, depletion of CBP and p300 impaired the camptothecin (CPT)- and neocarzinostatin (NCS)-induced phosphorylation of RPA32 (H1299 cells in **Figure** **4A** and HeLa cells in **Figure** **S3A**). As in the case of BRCA1 depletion, knockdown of CBP and p300 also decreased the levels of RPA32 and RPA70 proteins in the chromatin fraction of CPT-treated cells, indicating that their recruitment to damaged chromatin had been impaired (**Figure** **4B–C**). Similar results were obtained in cells subjected to NCS- and IR-induced DNA damage (**Figure** **4D–E**). In addition, the proportion of cells positive for CPT-induced RPA32 and phosphorylated RPA32 foci was significantly reduced upon double knockdown of CBP and p300 (H1299 cells in **Figure** **4F–G** and HeLa cells in **Figure** **S3B–C**). The formation of CPT- and IR-induced BRCA1 foci was also impaired upon depletion of CBP and p300 (H1299 cells in **Figure** **4H–I** and HeLa cells in **S3D–E**). Thus, knockdown of CBP and p300 results in BRCA1 deficiency, which in turn reduces the generation of ssDNA.

The BRCA1-CtIP-MRN complex promotes the processing of DSBs to generate RPA-coated ssDNA, which is required for the activation of ATR [8], [9], [10], [13]. Consistent with this, depletion of CBP and p300 impaired CHK1 phosphorylation after DNA damage without significantly affecting the phosphorylation status of ATM and CHK2 (**Figure** **4A**), indicating the impairment of ATR-CHK1 but not ATM-CHK2 signaling. ATR-CHK1 signaling regulates HR as well as the DNA damage checkpoint [13]. Therefore, we next examined whether the depletion of CBP and p300 abrogates the DNA damage checkpoint. IR treatment reduced the proportion of cells positive for phosphorylated histone H3 (Ser10), a marker of mitosis, indicating the induction of cell cycle arrest at the G2 phase via activation of the G2/M DNA damage checkpoint. This reduction was impaired upon depletion of CBP and p300 in H1299, HeLa, and A549 cells (**Figure** **4J** **and Figure** **S4A–B**). These results indicate that the depletion of CBP and p300 impairs not only HR but also the DNA damage checkpoint by suppressing ATR-CHK1 signaling. This result is supported by the observation that CBP- and p300-depleted cells are more sensitive than control cells to CPT (**Figure** **4K, S** **4C–D**).

## Discussion

Previously, we showed that CBP and p300 are recruited to DSB sites, where they acetylate histones H3 and H4 [4]. In this study, we demonstrated that CBP and p300 play an additional role in DSB repair, namely the transcriptional regulation of *BRCA1* and *RAD51*, two key genes involved in HR, through acetylation of histones H3 and H4 at the promoter regions of these two genes. These results suggest that DSB-induced HR is regulated by the HAT activities of CBP and p300 in two ways: histone acetylation at promoter regions of HR-related genes, which up-regulates and maintains HR protein expression, and histone acetylation at DSB sites, which allows recruitment of proteins required for HR repair. By functioning as co-activators, CBP and p300 act as hubs in several gene regulatory networks and thereby regulate a variety of intracellular processes [31]. We have shown here that in HR, these HATs activate transcription by regulating the accessibility of the transcription factors E2F1 and E2F4. This regulation of *BRCA1* and *RAD51* in HR contrasts with the regulation of factors involved in NHEJ, the other major DSB repair system, in which CBP and p300 are not involved in transcriptional activation of key genes [4] (**Figure** **2A**). Thus, the major pathways involved in DSB repair are likely to be regulated in different ways by CBP and p300 HATs.

We have shown here that *BRCA1* and *RAD51* are transcriptionally regulated by CBP and p300. BRCA1 has multiple functions in several DNA repair pathways and the DNA damage checkpoint, whereas RAD51 exclusively functions in HR [32]. Therefore, CBP and p300, which affect transcription of both the *BRCA1* and *RAD51* genes, may be important for both DSB repair and the DNA damage checkpoint. One of the functions of the BRCA1 protein is activation of a DNA end resection complex. Indeed, in this study, we showed that downregulation of BRCA1 upon depletion of CBP and p300 impairs the generation of ssDNA essential for RPA binding, leading to abrogation of HR and ATR-CHK1 signaling. Depletion of CBP and p300 impaired the activation of the G2/M DNA damage checkpoint associated with suppression of ATR-CHK1 signaling. On the other hand, CBP and p300 also contribute to the intra-S checkpoint through their interactions with ATR [33]. Therefore, CBP and p300 might have functions in multiple checkpoint pathways. Our results showed that CHK1 protein expression was reduced upon depletion of CBP and p300 (**Figure** **4A**), although we did not investigate the mechanism underlying this reduction. Thus, impairment of the G2/M DNA damage checkpoint by CBP and p300 ablation might be due not only to the suppression of the BRCA1-mediated DNA damage response pathway, but also to the suppression of *CHK1* expression. Further studies are needed to elucidate the mechanisms by which the DNA damage checkpoint is regulated by CBP and p300.

CBP and p300 proteins acetylate histones and non-histone proteins by interacting with hundreds of proteins and thereby regulate diverse intra-nuclear processes [31]. Here, we have presented a piece of evidence that these two HATs play an important role in DSB repair and the DNA damage checkpoint, which are critical for the maintenance of genome homeostasis, through transcriptional activation of the *RAD51* and *BRCA1* genes. Notably, CBP and p300 have also been shown to be critical for cell proliferation [34], [35], although such effects were not observed in association with short term CBP and p300 depletion in the present study (**Figure** **2D**). Their role in the maintenance of genome homeostasis might also facilitate constitutive cell proliferation; however, further functional studies are necessary to understand fully how the CBP/p300 and DSB repair and DNA damage checkpoint machineries are integrated to function harmoniously together.

## Supporting Information

Figure S1 **Involvement of CBP and p300 in HR.** (**A**) Suppression of I-*Sce*I-induced HR repair upon CBP and p300 depletion. Representative flow cytometry images for the experiments in Figure 1B are shown. (**B**) Immunoblots of whole cell lysates of cells harvested 48 hr after transfection with the indicated siRNAs. (**C**) CPT-induced formation of RAD51 foci. A549 cells were transfected with non-targeting (siNT) or CBP and p300 (siC+p) siRNA for 48 hr, treated with DMSO (NT) or 1 μM camptothecin (CPT) for 4 hr, and then analyzed by immunofluorescence. The percentages of cells with >10 RAD51 foci are shown. Data represent the mean ± SD. (**D, E**) IR-induced formation of RAD51 foci. H1299 or HeLa cells were transfected with non-targeting (siNT) or CBP and p300 (siC+p) siRNA for 48 hr, treated with 10 Gy ionizing irradiation (IR) for 4 hr or not irradiated (NT), and analyzed by immunofluorescence. Percentages of cells with >10 RAD51 foci are shown. Data represent the mean ± SD.
(TIF)

Figure S2 **Cell cycle profiles after CBP and p300 depletion.** (**A, B**) Down-regulation of BRCA1 and RAD51 proteins upon depletion of CBP and/or p300. Cells (A, HeLa; B, A549) were transfected with non-targeting (siNT) or CBP+p300 (siC+p) siRNAs for 48 hr and analyzed by immunoblotting. (**C–F**) Reduction of *BRCA1* and *RAD51* transcripts in CBP- and p300-depleted cells. Cells (C, E, HeLa; D, F, A549) were transfected with non-targeting (siNT) or CBP+p300 (siC+p) siRNAs for 48 hr and analyzed by quantitative real-time PCR for the detection of *BRCA1* (C, D) and *RAD51* (E, F) mRNAs. Expression levels were normalized against the levels of *GAPDH* mRNA. Data represent the mean ± SD.
(TIF)

Figure S3 **Impairment of RPA foci formation and checkpoint activation in CBP and p300 depleted cells.** (**A**) Effects of CBP and p300 depletion on DSB-induced RPA and CHK1 activation. HeLa cells were transfected with non-targeting (siNT) or CBP and p300 (siC+p) siRNA for 48 hr, treated with DMSO (NT) or 500 ng/ml neocarzinostatin (NCS) for 2 hr, and whole cell extracts were subjected to immunoblot analysis. (**B, C**) Decreased DSB-induced RPA32 foci formation. HeLa cells were transfected with non-targeting (siNT) or CBP and p300 (siC+p) siRNA for 48 hr, treated with DMSO (NT) or 1 μM camptothecin (CPT) for 3 hr, and subjected to immunofluorescence analysis. The percentages of cells with >10 RPA32 foci (B) or phosphorylated RPA32 (pS4/S8) foci (C) are shown. Data represent the mean ± SD. (**D, E**) CPT- and IR-induced formation of BRCA1 foci. HeLa cells were transfected with non-targeting (siNT) or CBP and p300 (siC+p) siRNA for 48 hr, treated with 1 μM camptothecin (CPT) for 3 hr (D) or 10 Gy ionizing irradiation (IR) for 4 hr (E), and then subjected to immunofluorescence analysis. The percentages of cells with >10 BRCA1 foci are shown. Data represent the mean ± SD.
(TIF)

Figure S4 **Impairment of DNA damage checkpoint activation and sensitivity to camptothecin in CBP and p300 depleted cells.** (**A, B**) Suppression of the G2/M DNA damage checkpoint. HeLa (A) or A549 (B) cells were transfected with non-targeting (siNT) or CBP and p300 (siC+p) siRNA for 48 hr. The cells were then irradiated (IR, 3 Gy) or not irradiated (No IR) and, after 1 hr, they were stained with anti-phospho-histone H3 antibody and propidium iodide (PI). The percentage of mitotic cells (mitotic index) was determined by flow cytometry. Data represent the mean ± SD. (**C, D**) Colony formation of HeLa or A549 cells treated with CPT. HeLa (C) or A549 (D) cells were transfected with non-targeting (siNT) or CBP and p300 (siC+p) siRNA for 48 hr. siRNA-treated cells were replated and incubated for 10–14 days. Survival is expressed as a percentage representing the number of colonies in treated samples relative to the number in DMSO-treated samples. Data represent the mean ± SD.
(TIF)
