# Supplementary figures and images for: CBP and p300 Histone Acetyltransferases Contribute to Homologous Recombination by Transcriptionally Activating the BRCA1 and RAD51 Genes
Source: PLoS One. 2012 Dec 20;7(12):e52810. doi: 10.1371/journal.pone.0052810 (PMC3527616; doi:10.1371/journal.pone.0052810)

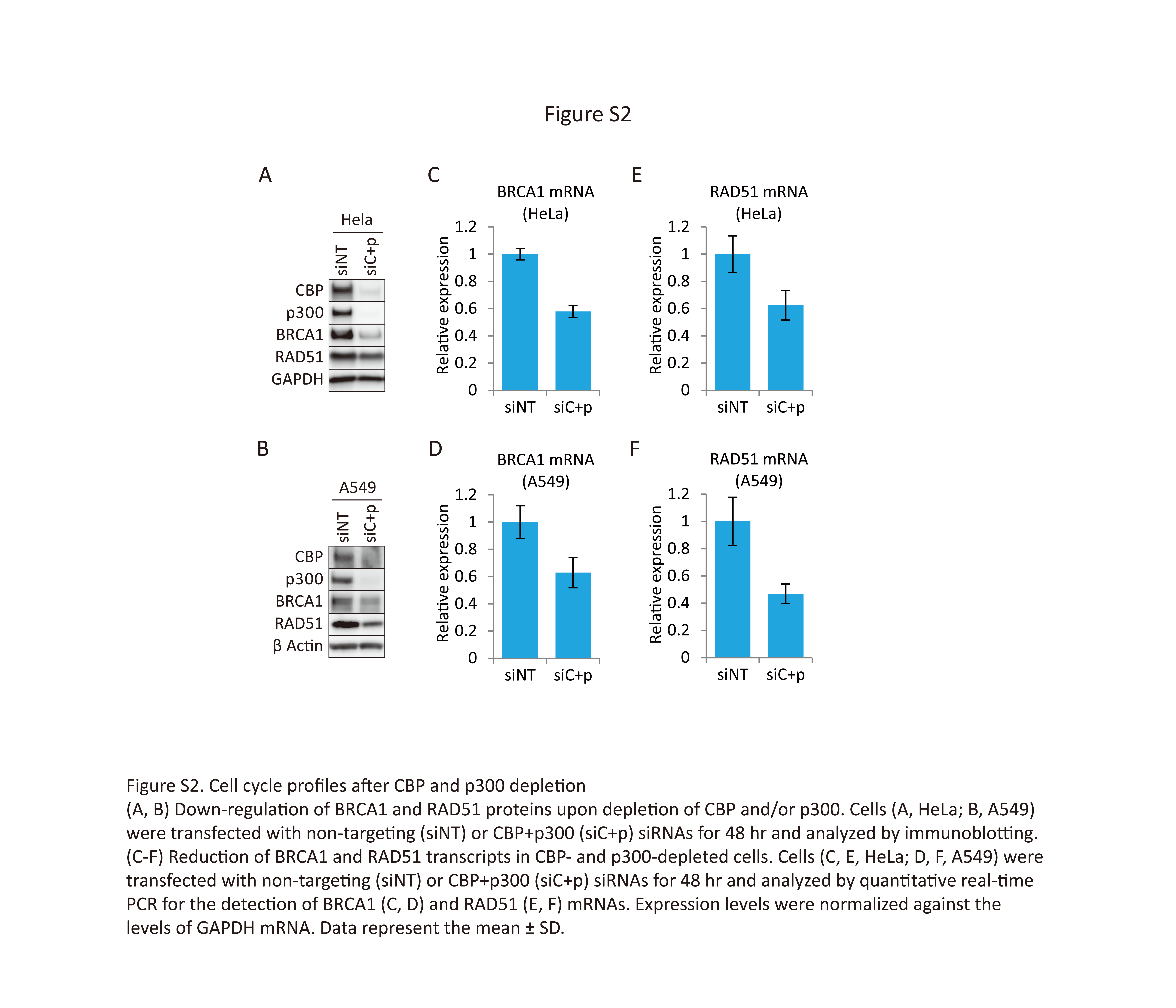

Supplement: Figure S2 — Cell cycle profiles after CBP and p300 depletion. (A, B) Down-regulation of BRCA1 and RAD51 proteins upon depletion of CBP and/or p300. Cells (A, HeLa; B, A549) were transfected with non-targeting (siNT) or CBP+p300 (siC+p) siRNAs for 48 hr and analyzed by immunoblotting. (C–F) Reduction of BRCA1 and RAD51 transcripts in CBP- and p300-depleted cells. Cells (C, E, HeLa; D, F, A549) were transfected with non-targeting (siNT) or CBP+p300 (siC+p) siRNAs for 48 hr and analyzed by quantitative real-time PCR for the detection of BRCA1 (C, D) and RAD51 (E, F) mRNAs. Expression levels were normalized against the levels of GAPDH mRNA. Data represent the mean ± SD. (TIF) [file pone.0052810.s002.tif]

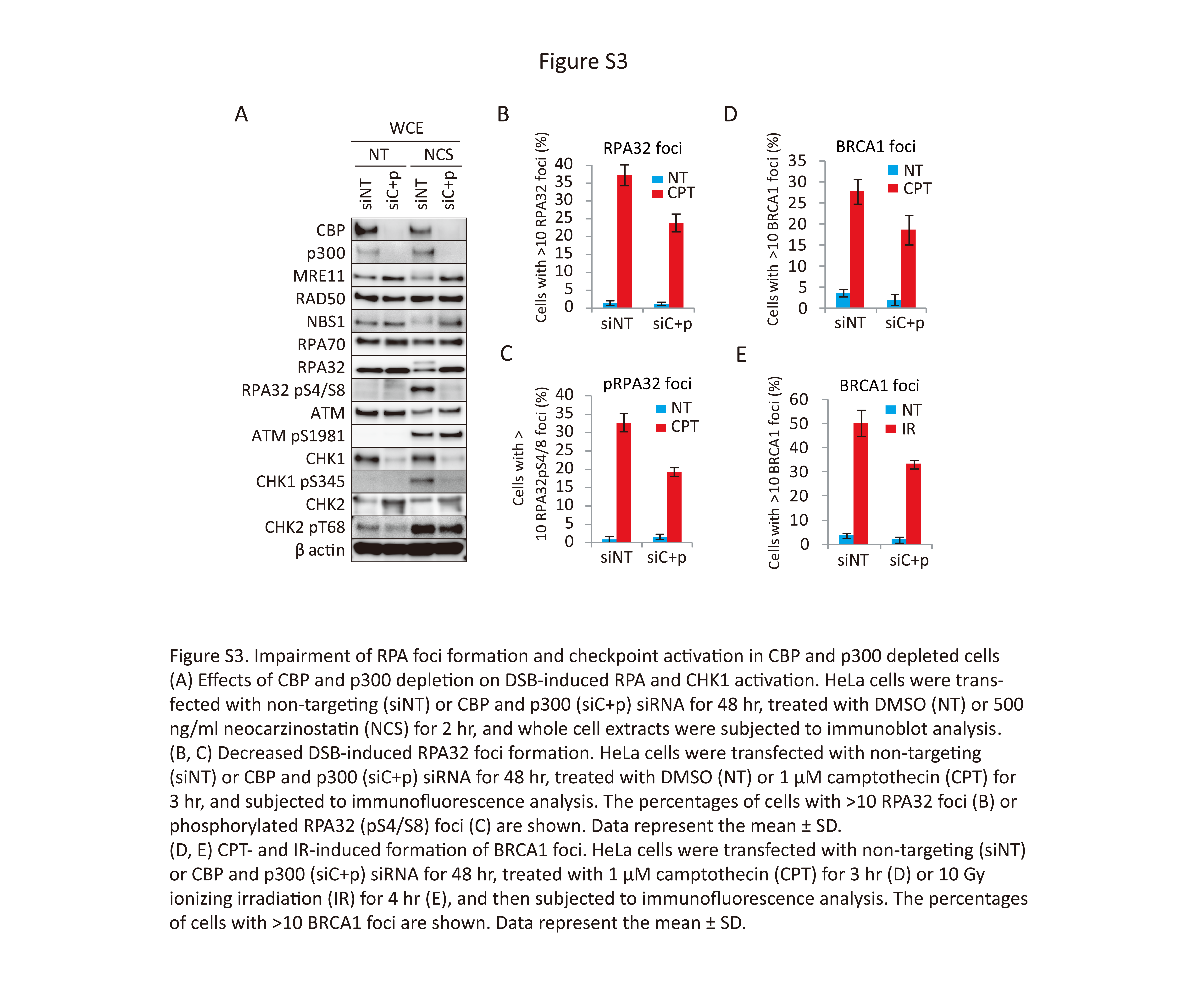

Supplement: Figure S3 — Impairment of RPA foci formation and checkpoint activation in CBP and p300 depleted cells. (A) Effects of CBP and p300 depletion on DSB-induced RPA and CHK1 activation. HeLa cells were transfected with non-targeting (siNT) or CBP and p300 (siC+p) siRNA for 48 hr, treated with DMSO (NT) or 500 ng/ml neocarzinostatin (NCS) for 2 hr, and whole cell extracts were subjected to immunoblot analysis. (B, C) Decreased DSB-induced RPA32 foci formation. HeLa cells were transfected with non-targeting (siNT) or CBP and p300 (siC+p) siRNA for 48 hr, treated with DMSO (NT) or 1 μM camptothecin (CPT) for 3 hr, and subjected to immunofluorescence analysis. The percentages of cells with >10 RPA32 foci (B) or phosphorylated RPA32 (pS4/S8) foci (C) are shown. Data represent the mean ± SD. (D, E) CPT- and IR-induced formation of BRCA1 foci. HeLa cells were transfected with non-targeting (siNT) or CBP and p300 (siC+p) siRNA for 48 hr, treated with 1 μM camptothecin (CPT) for 3 hr (D) or 10 Gy ionizing irradiation (IR) for 4 hr (E), and then subjected to immunofluorescence analysis. The percentages of cells with >10 BRCA1 foci are shown. Data represent the mean ± SD. (TIF) [file pone.0052810.s003.tif]

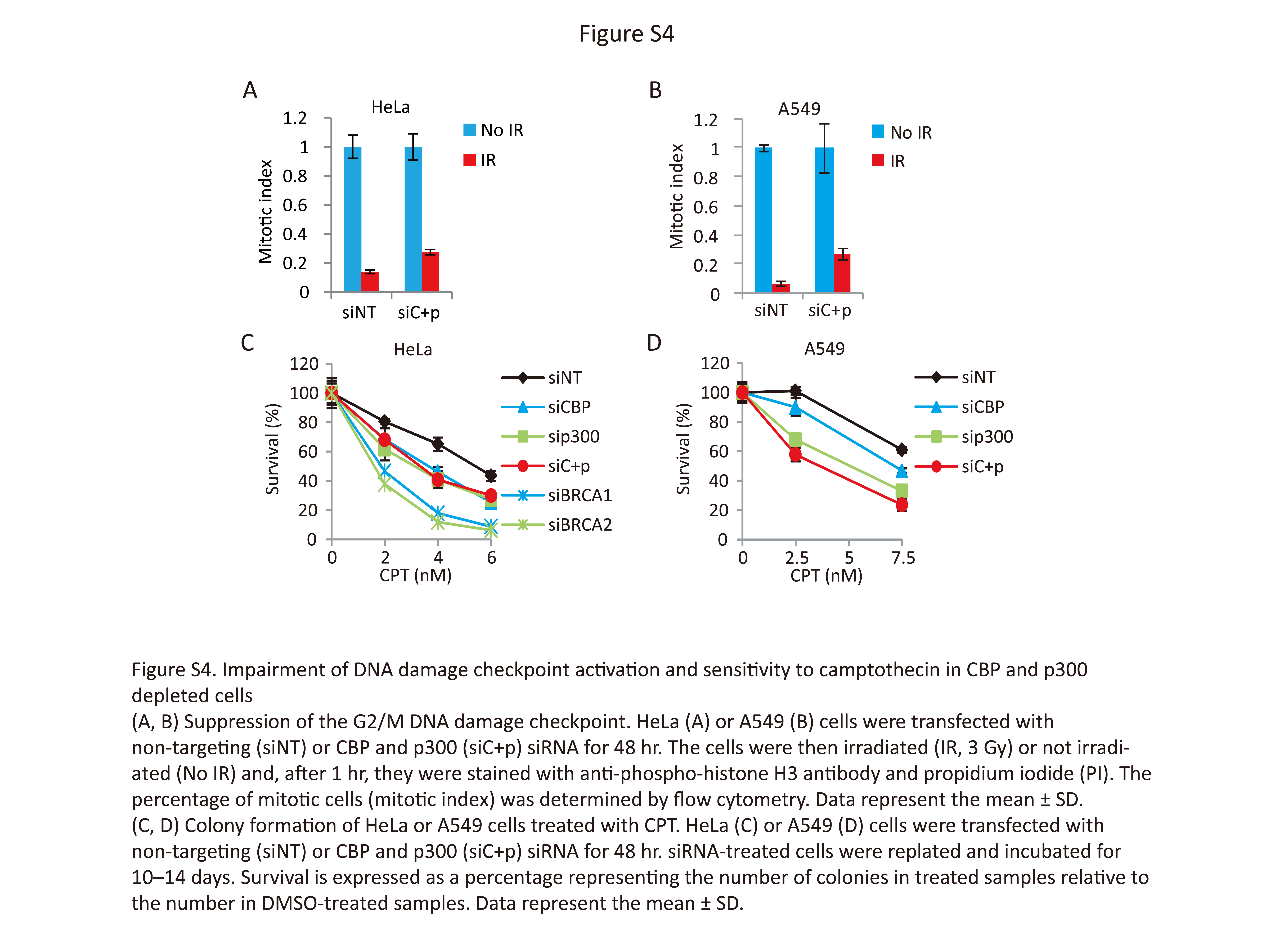

Supplement: Figure S4 — Impairment of DNA damage checkpoint activation and sensitivity to camptothecin in CBP and p300 depleted cells. (A, B) Suppression of the G2/M DNA damage checkpoint. HeLa (A) or A549 (B) cells were transfected with non-targeting (siNT) or CBP and p300 (siC+p) siRNA for 48 hr. The cells were then irradiated (IR, 3 Gy) or not irradiated (No IR) and, after 1 hr, they were stained with anti-phospho-histone H3 antibody and propidium iodide (PI). The percentage of mitotic cells (mitotic index) was determined by flow cytometry. Data represent the mean ± SD. (C, D) Colony formation of HeLa or A549 cells treated with CPT. HeLa (C) or A549 (D) cells were transfected with non-targeting (siNT) or CBP and p300 (siC+p) siRNA for 48 hr. siRNA-treated cells were replated and incubated for 10–14 days. Survival is expressed as a percentage representing the number of colonies in treated samples relative to the number in DMSO-treated samples. Data represent the mean ± SD. (TIF) [file pone.0052810.s004.tif]
